# Natural language processing for humanitarian action: Opportunities, challenges, and the path toward humanitarian NLP

**DOI:** 10.3389/fdata.2023.1082787

**Published:** 2023-03-24

**Authors:** Roberta Rocca, Nicolò Tamagnone, Selim Fekih, Ximena Contla, Navid Rekabsaz

**Affiliations:** ^1^Department of Culture, Cognition and Computation, Aarhus University, Aarhus, Denmark; ^2^Data Friendly Space, Richmond, VA, United States; ^3^Institute of Computational Perception, Johannes Kepler University, Linz, Austria; ^4^Linz Institute of Technology, AI Lab, Linz, Austria

**Keywords:** NLP, humanitarian response, machine learning, transformers, social good

## Abstract

Natural language processing (NLP) is a rapidly evolving field at the intersection of linguistics, computer science, and artificial intelligence, which is concerned with developing methods to process and generate language at scale. Modern NLP tools have the potential to support humanitarian action at multiple stages of the humanitarian response cycle. Both internal reports, secondary text data (e.g., social media data, news media articles, or interviews with affected individuals), and external-facing documents like Humanitarian Needs Overviews (HNOs) encode information relevant to monitoring, anticipating, or responding to humanitarian crises. Yet, lack of awareness of the concrete opportunities offered by state-of-the-art techniques, as well as constraints posed by resource scarcity, limit adoption of NLP tools in the humanitarian sector. This paper provides a pragmatically-minded primer to the emerging field of humanitarian NLP, reviewing existing initiatives in the space of humanitarian NLP, highlighting potentially impactful applications of NLP in the humanitarian sector, and describing criteria, challenges, and potential solutions for large-scale adoption. In addition, as one of the main bottlenecks is the lack of data and standards for this domain, we present recent initiatives (the DEEP and HumSet) which are directly aimed at addressing these gaps. With this work, we hope to motivate humanitarians and NLP experts to create long-term impact-driven synergies and to co-develop an ambitious roadmap for the field.

## 1. Introduction

Natural language processing (NLP) is a field at the intersection of linguistics, computer science, and artificial intelligence concerned with developing computational techniques to process and analyze text and speech. Owing to the increased availability of large-scale text data (e.g., social media data, news archives, web content) and to advances in computing infrastructure, NLP has witnessed rapid and dramatic developments over the past few years (Ruder, 2018b; Min et al., 2021). State-of-the-art language models can now perform a vast array of complex tasks, ranging from answering natural language questions to engaging in open-ended dialogue, at levels that sometimes match expert human performance. Open-source initiatives such as spaCy^1^ and Hugging Face's libraries (e.g., Wolf et al., 2020) have made these technologies easily accessible to a broader technical audience, greatly expanding their potential for application.

The humanitarian sector—that is, the ecosystem of organizations and activities aimed at providing assistance in the context of crises and disasters—could greatly benefit from tools that make it possible to draw operational insights from large volumes of text data. Humanitarian organizations produce large amounts of unstructured text data, for example in the form of internal reports that distill expert knowledge on specific crisis contexts and outline suitable response strategies, and external-facing documents like humanitarian need overviews (HNOs). Secondary sources such as news media articles, social media posts, or surveys and interviews with affected individuals also contain important information that can be used to monitor, prepare for, and efficiently respond to humanitarian crises. NLP techniques could help humanitarians leverage these source of information at scale to better understand crises, engage more closely with affected populations, or support decision making at multiple stages of the humanitarian response cycle. However, systematic use of text and speech technology in the humanitarian sector is still extremely sparse, and very few initiatives scale beyond the pilot stage.

Limited adoption of NLP techniques in the humanitarian sector is arguably motivated by a number of factors. First, high-performing NLP methods for unstructured text analysis are relatively new and rapidly evolving (Min et al., 2021), and their potential may not be entirely known to humanitarians. Secondly, the humanitarian sector still lacks the kind of large-scale text datasets and data standards required to develop robust domain-specific NLP tools. Data scarcity becomes an especially salient issue when considering that humanitarian crises often affect populations speaking low-resource languages (Joshi et al., 2020), for which little if any data is digitally available. Thirdly, it is widely known that publicly available NLP models can absorb and reproduce multiple forms of biases (e.g., racial or gender biases Bolukbasi et al., 2016; Davidson et al., 2019; Bender et al., 2021). Safely deploying these tools in a sector committed to protecting people in danger and to causing no harm requires developing solid *ad-hoc* evaluation protocols that thoroughly assess ethical risks involved in their use.

Overcoming these challenges and enabling large-scale adoption of NLP techniques in the humanitarian response cycle is not simply a matter of scaling technical efforts. It requires dialogue between humanitarian practitioners and NLP experts, as well as platforms for collaborative experimentation, where humanitarians' expert knowledge of real-world needs and constraints can inform the design of scalable technical solutions. To encourage this dialogue and support the emergence of an impact-driven humanitarian NLP community, this paper provides a concise, pragmatically-minded primer to the emerging field of humanitarian NLP. By providing a high-level introduction to modern NLP, identifying potential applications in the humanitarian sector, and discussing outstanding challenges and potential solutions, we hope to inspire humanitarians and NLP experts to engage in co-developing an ambitious roadmap for the field.

The paper is structured as follows. First, we provide a short primer to NLP (Section 2), and introduce foundational principles and defining features of the humanitarian world (Section 3). Secondly, we provide concrete examples of how NLP technology could support and benefit humanitarian action (Section 4). As we highlight in Section 4, lack of domain-specific large-scale datasets and technical standards is one of the main bottlenecks to large-scale adoption of NLP in the sector. This is why, in Section 5, we describe The Data Entry and Exploration Platform (DEEP^2^), a recent initiative (involving authors of the present paper) aimed at addressing these gaps.

Finally, we analyze and discuss the main technical bottlenecks to large-scale adoption of NLP in the humanitarian sector, and we outline possible solutions (Section 6). We conclude by highlighting how progress and positive impact in the humanitarian NLP space rely on the creation of a functionally and culturally diverse community, and of spaces and resources for experimentation (Section 7).

In line with its aim of inspiring cross-functional collaborations between humanitarian practitioners and NLP experts, the paper targets a varied readership and assumes no in-depth technical knowledge.

## 2. Natural language processing: A short primer

### 2.1. What is NLP?

Language is foundational to human societies. We produce language for a significant portion of our daily lives, in written, spoken or signed form, in natively digital or digitizable formats, and for goals that range from persuading others, to communicating and coordinating our behavior. The field of NLP is concerned with developing techniques that make it possible for machines to represent, understand, process, and produce language using computers. Being able to efficiently represent language in computational formats makes it possible to automate traditionally analog tasks like extracting insights from large volumes of text, thereby scaling and expanding human abilities.

### 2.2. Typical NLP tasks

NLP researchers focus on developing systems that can perform a range of analytical or predictive tasks. Traditional NLP problems include, for example: developing systems that can extract useful representations of the content of texts (e.g., for the purpose of automatically extracting the main “topics” expressed in a set of texts, a task known as *topic modeling*); developing tools that identify mentions of “named entities” (e.g., locations, organizations, people) in a text (*named entity recognition*); automatically extracting the sentiment of a given text (*sentiment classification*). *Automated text summarization* (automated generation of summaries from long texts), *machine translation* (automated translation of texts from a source to a target language), and *question answering* (automated generation or selection of answers to a given input question) are examples of more advanced problems which have especially benefited from recent advances in NLP methods. Speech-related tasks such as *speech recognition* (automated transcription of spoken language) and *speech synthesis* (automated text-to-audio synthesis) are also sometimes considered a part of NLP. While early NLP predominantly relied on rule-based or symbolic systems, modern NLP is almost entirely dominated by statistical and machine learning approaches, which deliver higher performance and provide greater cross-domain generalizability^3^.

### 2.3. Words as vectors: From rule-based to statistical NLP

**Figure 1:**
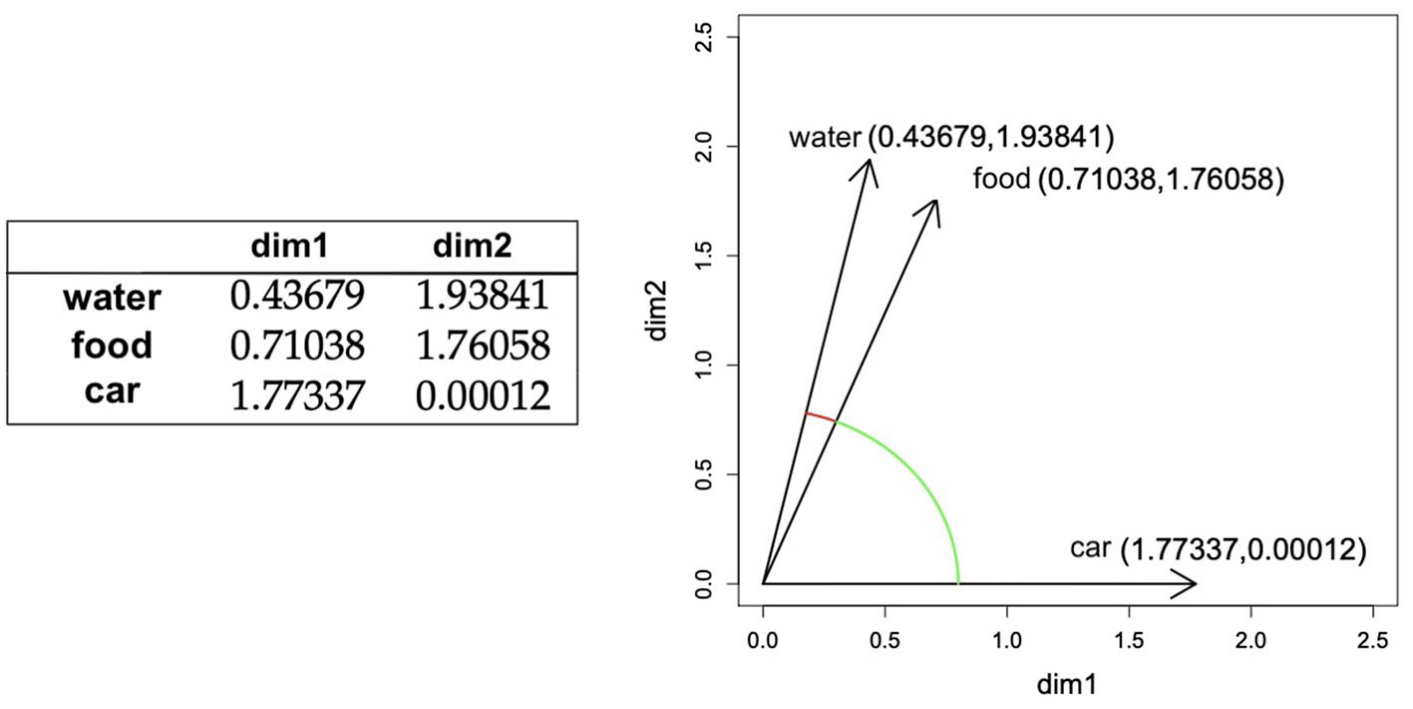
Toy example of distributional semantic representations, figure and caption from Boleda and Herbelot (2016), Figure 2, (with adaptations). On the left, a toy distributional semantic lexicon, with words being represented through 2-dimensional vectors. On the right, the representations of those words in semantic space. Semantic distance between words can be computed as geometric distance between their vector representations. Words with more similar meanings will be closer in semantic space than words with more different meanings. In this specific example, distance (see arcs) between vectors for *food* and *water* is smaller than the distance between the vectors for *water* and *car*.

*Distributional semantics* (Harris, 1954; Schütze, 1992; Landauer and Dumais, 1997) is one of the paradigms that has had the most impact on modern NLP, driving its transition toward statistical and machine learning-based approaches. Distributional semantics is grounded in the idea that the meaning of a word can be defined as the set of contexts in which the word tends to occur. Based on this assumption, words can be represented as vectors of numbers that quantify (more or less explicitly) how often they tend to co-occur with each other word in the vocabulary (i.e., being present in the same sentence, or in a window of given length). These vectors can be interpreted as coordinates on a high-dimensional semantic space where words with similar meanings (“cat” and “dog”) will be closer than words whose meaning is very different (“cat” and “teaspoon”, see Figure 1). This simple intuition makes it possible to represent the meaning of text in a quantitative form that can be operated upon algorithmically or used as input to predictive models. We refer to Boleda (2020) for a deeper explanation of this topic, and also to specific realizations of this idea under the word2vec (Mikolov et al., 2013), GloVe (Bojanowski et al., 2016), and fastText (Pennington et al., 2014) algorithms.

### 2.4. The deep learning era: Meet *transformers*

**Figure 2:**
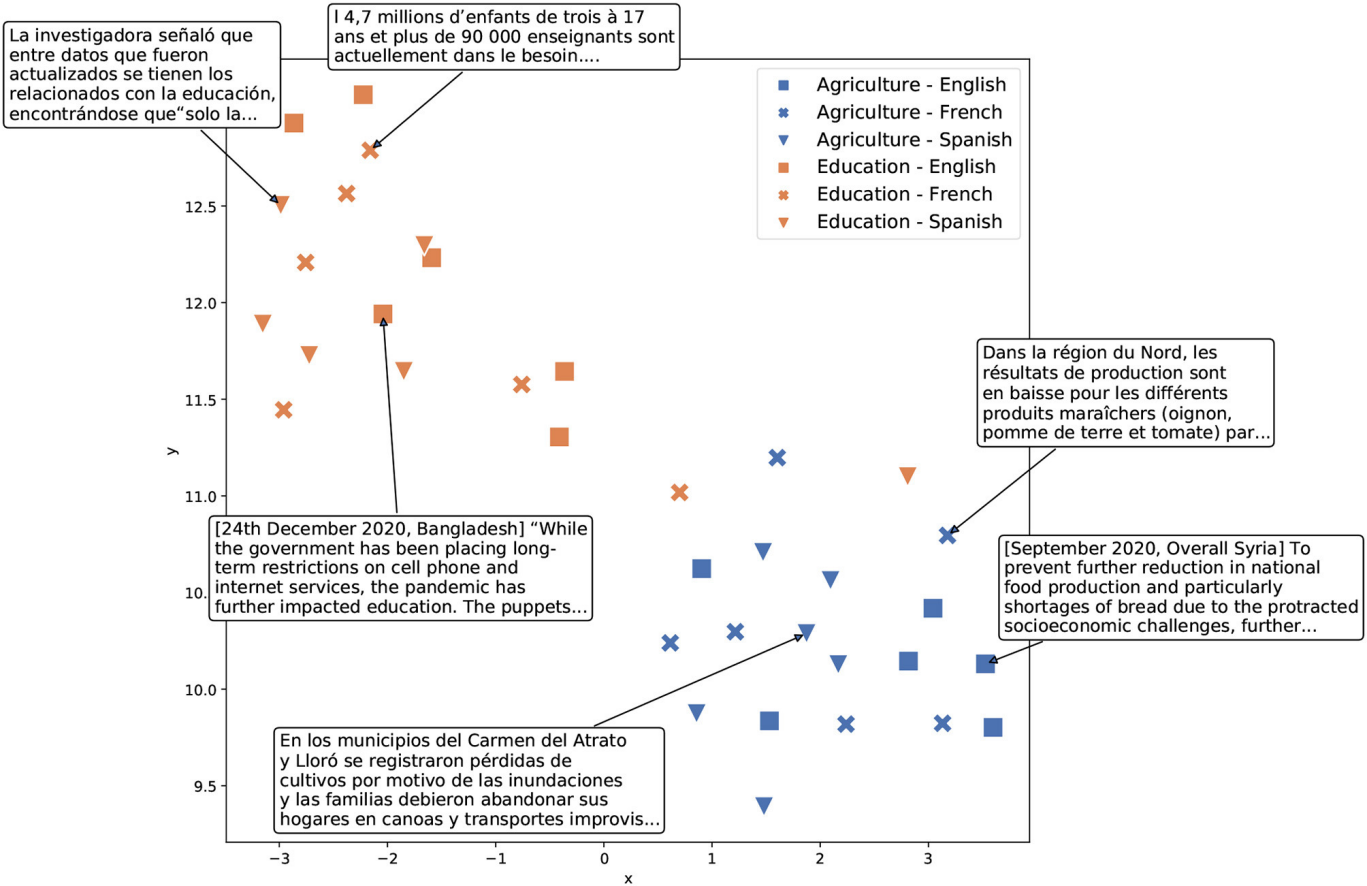
Vector representations of sample text excerpts in three languages created by the USE model, a multilingual transformer model, (Yang et al., 2020) and projected into two dimensions using TSNE (van der Maaten and Hinton, 2008). Text excerpts are extracted from a recent humanitarian response dataset (HumSet, Fekih et al., 2022; see Section 5 for details). As shown, the language model correctly separates the text excerpts about various topics (Agriculture vs. Education), while the excerpts on the same topic but in different languages appear in close proximity to each other.

Over the past few years, NLP has witnessed tremendous progress, with the advent of deep learning models for text and audio (LeCun et al., 2015; Ruder, 2018b; Young et al., 2018) inducing a veritable paradigm shift in the field^4^. Central to these recent advancements is the transformer architecture (Vaswani et al., 2017), which makes it possible to learn highly contextualized and semantically rich representations of language elements at the level of both individual words and text sequences. The transformer architecture has become the essential building block of modern NLP models, and especially of large language models such as BERT (Devlin et al., 2019), RoBERTa (Liu et al., 2019), and GPT models (Radford et al., 2019; Brown et al., 2020). These large neural networks are trained on simple predictive tasks such as reconstructing the missing word in an input sentence—*masked language modeling* (Devlin et al., 2019; Liu et al., 2019)—or guessing the next word given an input sequence—*forward language modeling* (Radford et al., 2019; Brown et al., 2020). Through these general pre-training tasks, language models learn to produce high-quality vector representations of words and text sequences, encompassing semantic subtleties, and linguistic qualities of the input. Individual language models can be trained (and therefore deployed) on a single language, or on several languages in parallel (Conneau et al., 2020; Minixhofer et al., 2022). To gain a better understanding of the semantic as well as multilingual aspects of language models, we depict an example of such resulting vector representations in Figure 2.

The vector representations produced by these language models can be used as inputs to smaller neural networks and fine-tuned (i.e., further trained) to perform virtually any downstream predictive tasks (e.g., sentiment classification). This powerful and extremely flexible approach, known as *transfer learning* (Ruder et al., 2019), makes it possible to achieve very high performance on many core NLP tasks with relatively low computational requirements. Importantly, platforms such as Hugging Face (Wolf et al., 2020) and SpaCy have made pretrained transformers trivial to access and to fine-tune on custom datasets and tasks, greatly increasing their impact and applicability across a virtually unlimited range of real-life contexts.

### 2.5. Limitations

While NLP systems achieve impressive performance on a wide range of tasks, there are important limitations to bear in mind. First, state-of-the-art deep learning models such as transformers require large amounts of data for pre-training. This data is hardly ever available for languages with small speaker communities, which results in high-performing models only being available for a very limited set of languages (Joshi et al., 2020; Nekoto et al., 2020). There is increasing awareness of these issues in the NLP community, and efforts are being undertaken to tackle this problem by developing more efficient models and training methods (Minixhofer et al., 2022), large multilingual models (see BigScience's BLOOM^5^ and Costa-jussà et al., 2022), and training resources for underrepresented languages (Lakew et al., 2020; Kemp, 2021).

Secondly, pretrained NLP models often absorb and reproduce biases (e.g., gender and racial biases) present in the training data (Shah et al., 2019; Blodgett et al., 2020). This is also a known issue within the NLP community, and there is increasing focus on developing strategies aimed at preventing and testing for such biases.

Finally, modern NLP models are “black boxes”; explaining the decision mechanisms that lead to a given prediction is extremely challenging, and it requires sophisticated *post-hoc* analytical techniques. This is especially problematic in contexts where guaranteeing accountability is central, and where the human cost of incorrect predictions is high.

## 3. The humanitarian world at a glance

### 3.1. Defining humanitarian action: Principles and ecosystem

Formulating a comprehensive definition of humanitarian action is far from straightforward. Broadly speaking, humanitarian action encompasses all activities aimed at “saving lives, protecting livelihoods, alleviating suffering, and maintaining human dignity during and in the aftermath of crisis,” or at “prevent[ing] and strengthen[ing] preparedness for the occurrence of such situations” (Maxwell and Gelsdorf, 2019).

Four foundational principles demarcate humanitarian action from other forms of protection or assistance: *humanity, impartiality, neutrality*, and *independence*. *Humanity* translates into the moral imperative to limit human suffering and protect human lives under any circumstances. *Impartiality* dictates the need to address suffering without discrimination. *Neutrality* and *independence* are more “operational” principles. The *neutrality* principle dictates that humanitarian aid should not favor any side in a conflict. *Independence* dictates that humanitarian action be “free from the coercion of states and political actors” (Maxwell and Gelsdorf, 2019, p. 10). Despite having been a source of controversy, these principles are foundational to humanitarian action, as they distinguish humanitarian aid from other forms of activities carried out on political, religious, or military grounds.

Humanitarian assistance can be provided in many forms and at different spatial (global and local) and temporal (before, during, and after crises) scales. As a result, the humanitarian world is a complex and highly dynamic ecosystem of actors and stakeholders, which revolves around affected populations and involves international organizations and humanitarian networks as well as local NGOs, peacekeeping actors, militaries, governments, and donors. The specifics of the humanitarian ecosystem and of its response mechanisms vary widely from crisis to crisis, but larger organizations have progressively developed fairly consolidated governance, funding, and response frameworks. In the interest of brevity, we will mainly focus on response frameworks revolving around the United Nations, but it is important to keep in mind that this is far from being an exhaustive account of how humanitarian aid is delivered in practice.

### 3.2. Needs assessment and the humanitarian response cycle

Planning, funding, and response mechanisms coordinated by United Nations' humanitarian agencies are organized in *sectors* and *clusters*. *Clusters* are groups of humanitarian organizations and agencies that cooperate to address humanitarian needs of a given type. *Sectors* define the types of needs that humanitarian organizations typically address, which include, for example, food security, protection, health. Most crises require coordinating response activities across multiple sectors and clusters, and there is increasing emphasis on devising mechanisms that support effective inter-sectoral coordination.

The cluster structure is central to understanding how the UN plans and fundraises for humanitarian response. Every year, comprehensive assessments of humanitarian needs are conducted for each sector and for all crisis and at-risk contexts. Needs assessments are performed at the country level, and the resulting assessments feed into Humanitarian Needs Overviews (HNOs), that is, documents that outline humanitarians' shared understanding of the impact and evolution of a crisis, and that help inform response planning by providing an in-depth analysis of the situation and of associated needs^6^. HNOs are used to develop a Humanitarian Response Plan (HRP), which lays out the strategic agenda for each cluster and informs fundraising campaigns.

### 3.3. Using data for assessment and response

Sources feeding into needs assessments can range from qualitative interviews with affected populations to remote sensing data or aerial footage. Needs assessment methodologies are to date loosely standardized, which is in part inevitable, given the heterogeneity of crisis contexts. Nevertheless, there is increasing pressure toward developing robust and strongly evidence-based needs assessment procedures. *Anticipatory action* is also becoming central to the debate on needs assessment methodologies, and the use of predictive modeling to support planning and anticipatory response is gaining traction.

Pressure toward developing increasingly evidence-based needs assessment methodologies has brought data and quantitative modeling techniques under the spotlight. Over the past few years, UN OCHA's Centre for Humanitarian Data^7^ has had a central role in promoting progress in this domain. The Center has been working toward addressing fundamental gaps in the humanitarian data ecosystem by creating a platform for data sharing (HDX^8^), developing data annotation standards (HXL^9^), promoting data literacy and responsibility, and supporting predictive modeling approaches to response and anticipatory action.

The data and modeling landscape in the humanitarian world is still, however, highly fragmented. Datasets on humanitarian crises are often hard to find, incomplete, and loosely standardized. Even when high-quality data are available, they cover relatively short time spans, which makes it extremely challenging to develop robust forecasting tools. Text data are an outstanding example of this. While modern NLP methods have a vast potential to simplify and enhance stages of the humanitarian response cycle that range from assessment to decision-making, text data are hardly ever available in formats and scales that allow for computational analysis, and the humanitarian sector lags behind in reaping the benefits of recent NLP progress.

## 4. Developing a vision for humanitarian NLP

### 4.1. How can NLP support humanitarian response?

NLP is a rapidly advancing field, and sparse adoption of NLP technologies in the humanitarian sector may in part be due by limited awareness of their potential. Before discussing more technical aspects of humanitarian NLP it is therefore necessary to address some fundamental questions: what can NLP *concretely* do for humanitarians? Which stages of humanitarian planning and response can NLP support, and how?

Humanitarian organizations produce and process vast amounts of text. Large volumes of technical reports are produced on a regular basis, which convey factual information or distill expert knowledge on humanitarian crises. Interviews, surveys, and focus group discussions are conducted regularly to better understand the needs of affected populations. Alongside these “internal” sources of linguistic data, social media data and news media articles also convey information that can be used to monitor and better understand humanitarian crises. People make extensive use of social media platforms like Twitter and Facebook in the context of natural catastrophes and complex political crises, and news media often convey information on crisis-related drivers and events.

As discussed in Section 2, NLP techniques make it possible to synthesize and draw insights from large amounts of text. State-of-the-art methods even support automatic high-quality generation of fluent text from structured prompts and automatic translation, which can help streamline internal processes and enhance communication within and beyond organizations. Leveraging these capabilities, current NLP technology can be used to support evidence-based decision-making and facilitate humanitarian operations in a number of ways. Here, we highlight three potential application contexts in which we envisage a high potential for impact, and that can concretely be supported by existing technology:

- Tracking external data sources to monitor, predict, and understand the dynamics of crises;
- Enhancing communication with affected populations;
- Generating structured datasets from unstructured text.

### 4.2. Tracking external data sources to anticipate, monitor and understand crises

Social media posts and news media articles may convey information which is relevant to understanding, anticipating, or responding to both sudden-onset and slow-onset crises. The COVID-19 pandemic has been an outstanding example of this. Since the start of the pandemic, social media users and news media have constantly reported information relevant to tracking the spread and incidence of the disease, as well as information on its symptomatology—a type of bottom-up input that is especially relevant in the early stages of an emergency where medical knowledge is sparse and hard to access.

The use of social media data during the 2010 Haiti earthquake is an example of how social media data can be leveraged to map disaster-struck regions and support relief operations during a sudden-onset crisis (Meier, 2015). On January 12th, 2010, a catastrophic earthquake struck Haiti, causing widespread devastation and damage, and leading to the death of several hundred thousand people. In the immediate aftermath of the earthquake, a group of volunteers based in the United States started developing a “crisis map” for Haiti, i.e., an online digital map pinpointing areas hardest hit by the disaster, and flagging individual calls for help. Using Ushahidi^10^, a free online mapping platform that makes it possible to collect tweets and SMS sent to helplines in a keyword-based fashion, volunteers started tracking messages related to the Haiti disaster in real time, and to place them onto a map, either based on explicit mentions of locations, or by triangulating information with data from Google Earth and OpenStreetMap^11^. This resource, developed remotely through crowdsourcing and automatic text monitoring, ended up being used extensively by agencies involved in relief operations on the ground. While at the time mapping of locations required intensive manual work, current resources (e.g., state-of-the-art named entity recognition technology) would make it significantly easier to automate multiple components of this workflow.

The rise of “digital humanitarianism” (Meier, 2015) during the Haiti earthquake is a prominent example of how text data can be used to extract “actionable insights” (Castillo, 2016) in acute phases of a crisis, and directly support relief operations. But social media texts can also be used to gain more high-level situational awareness on ongoing crises or at-risk contexts. Social and news media can be used to track areas of public discourse relevant to identifying and monitoring risk factors associated with long-onset, political crises. This is, to the best of our knowledge, a hitherto unexplored space. For many social and news media, there are publicly available APIs which significantly simplify access to relevant data sources—though with some important exceptions^12^. Existing NLP resources like pretrained language models and topic modeling techniques provide a backbone of technical tools suitable to supporting these applications (Qadir et al., 2016).

There are a number of additional resources that are relevant to this class of applications. CrisisBench is a benchmark dataset including social media text labeled along dimensions relevant for humanitarian action (Alam et al., 2021). This dataset contains collections of tweets from multiple major natural disasters, labeled by relevance, intent (offering vs. requesting aid), and sector of interest. Classification models achieving high performance on this benchmark could be leveraged to filter social media content in real time, and provide humanitarians with additional sources of situational awareness (Vieweg et al., 2014; Imran et al., 2016, 2017; Kreutzer et al., 2019; Padhee et al., 2020; Lai et al., 2022). Lacuna Fund^13^ is an initiative that aims at increasing availability of unbiased labeled datasets from low- or middle-income contexts. Lacuna Fund mobilizes funding to collect datasets that can meet the needs of data and machine learning professionals working on developing analytical and predictive tools for underserved populations, and while the initiative is not specific to text data, NLP is one of its main focus areas^14^. Tools like AIDR (Imran et al., 2014) and MicroMappers—a platform and a crowdsourcing initiative for collection and annotation of social media datasets—were created with the intent of supporting social media analysis for humanitarian applications, but they are no longer maintained. On the other hand, Ushahidi is still actively maintained.

For further reference, similar projects in this space include IDMC's monitoring platform^15^, which extracts displacement-related information from GDELT and the European Media Monitor, HealthMap^16^, which scrapes web content to identify health-related news and alerts, and WHO's Epidemic Intelligence from Open Sources, a large-scale collaboration aimed at creating a unified approach to early detection, verification, assessment, and communication of public health threats from open data^17^.

Note, however, that the initiatives mentioned in the present section are fairly unique in the humanitarian world, and do not reflect a systematic effort toward large-scale implementation of NLP-driven technology in support of humanitarian monitoring and response.

### 4.3. Empowering communication with affected populations

Modeling tools similar to those deployed for social and news media analysis can be used to extract bottom-up insights from interviews with people at risk, delivered either face-to-face or via SMS and app-based chatbots. Using NLP tools to extract structured insights from bottom-up input could not only increase the precision and granularity of needs assessment, but also promote inclusion of affected individuals in response planning and decision-making.

Structured data collection technologies are already being used by humanitarian organizations to gather input from affected people in a distributed fashion. Technologies such as the KoBo Toolbox^18^ have been developed for this purpose. UNICEF's U-Report is an initiative inspired by a similar intent^19^. Modern NLP techniques would make it possible to expand these solutions to less structured forms of input, such as naturalistic text or voice recordings.

The potential of remote, text-based needs assessment is especially apparent for hard-to-reach contexts (e.g., areas where transportation infrastructure has been damaged), where it is impossible to conduct structured in-person interviews. In cases where affected individuals still retain access to digital technologies, NLP tools for information extraction or topic modeling could be used to process unstructured reports sent through SMS either spontaneously or through semi-structured prompts. This workflow could not only replace and streamline traditional structured assessments, but also help humanitarians extract novel information which is spontaneously provided by affected individuals, and may not otherwise be captured when following the strict constraints of highly structured interviews (see Kreutzer et al., 2019).

Remote devices, chatbots, and Interactive Voice Response systems (Bolton, 2018) can be used to track needs and deliver support to affected individuals in a personalized fashion, even in contexts where physical access may be challenging. A perhaps visionary domain of application is that of personalized health support to displaced people. It is known that speech and language can convey rich information about the physical and mental health state of individuals (see e.g., Rude et al., 2004; Eichstaedt et al., 2018; Parola et al., 2022). Both structured interactions and spontaneous text or speech input could be used to infer whether individuals are in need of health-related assistance, and deliver personalized support or relevant information accordingly.

Chatbots have previously been used to provide individuals with health-related assistance in multiple contexts^20^, and the Covid-19 pandemic has further accelerated the development of digital tools that can be deployed in the context of health emergencies. The use of language technology to deliver personalized support is, however, still rather sparse and unsystematic, and it is hard to assess the impact and scalability of existing applications.

A major challenge for these applications is the scarce availability of NLP technologies for small, low-resource languages. In displacement contexts, or when crises unfold in linguistically heterogeneous areas, even identifying which language a person in need is speaking may not be trivial. Here, language technology can have a significant impact in reducing barriers and facilitating communication between affected populations and humanitarians. To overcome the issue of data scarcity and support automated solutions to language detection and machine translation, Translators Without Borders (TWB) has launched a number of initiatives aimed at developing datasets and models for low-resource languages. One example is Gamayun (Öktem et al., 2020), a project aimed at crowdsourcing data from underrepresented languages. In a similar space is *Kató speak*, a voice-based machine translation model deployed during the 2018 Rohingya crisis.

There are a number of additional open-source initiatives aimed at contributing to improving NLP technology for underresourced languages. Mozilla Common Voice is a crowd-sourcing initiative aimed at collecting a large-scale dataset of publicly available voice data^21^ that can support the development of robust speech technology for a wide range of languages. Tatoeba^22^ is another crowdsourcing initiative where users can contribute sentence-translation pairs, providing an important resource to train machine translation models. Recently, Meta AI has released a large open-source machine translation model supporting direct translation between 200 languages, including a number of low-resource languages like Urdu or Luganda (Costa-jussà et al., 2022). Finally, Lanfrica^23^ is a web tool that makes it easy to discover language resources for African languages.

### 4.4. Generating structured datasets from unstructured text

NLP techniques can also be used to automate information extraction, e.g., by summarizing large volumes of text, extracting structured information from unstructured reports, or generating natural language reports from structured data (Yela-Bello et al., 2021; Fekih et al., 2022).

Tasks like named entity recognition (briefly described in Section 2) or relation extraction (automatically identifying relations between given entities) are central to these applications. For some domains (e.g., scientific and medical texts), domain-specific tools haven been developed that facilitate structured information extraction (see, for example scispaCy for biomedical text^24^), and similar tools could highly benefit the humanitarian sector. For example, while humanitarian datasets with rich historical data are often hard to find, reports often include the kind of information needed to populate structured datasets. Developing tools that make it possible to turn collections of reports into structured datasets automatically and at scale may significantly improve the sector's capacity for data analysis and predictive modeling.

There is increasing emphasis on developing models that can dynamically predict fluctuations in humanitarian needs, and simulate the impact of potential interventions. Being able to anticipate the unfolding of an epidemic over time (e.g., tracking cholera cases in affected areas), for example, and choosing the best type of intervention would be tremendously valuable, but doing so requires estimating models that capture dynamic causal relations between drivers of contagion (e.g., lack of sanitation, poor nutrition) and potential interventions (e.g., vaccinations, improvements in sanitation infrastructure, educational interventions) (Rocca, 2022). This, in turn, requires epidemiological data and data on previous interventions which is often hard to find in a structured, centralized form. Yet, organizations often issue written reports that contain this information, which could be converted into structured datasets using NLP technology.

Interestingly, NLP technology can also be used for the opposite transformation, namely generating text from structured information. Generative models such as models of the GPT family could be used to automatically produce fluent reports from concise information and structured data. An example of this is Data Friendly Space's experimentation with automated generation of Humanitarian Needs Overviews^25^. Note, however, that applications of natural language generation (NLG) models in the humanitarian sector are not intended to fully replace human input, but rather to simplify and scale existing processes. While the quality of text generated by NLG models is increasing at a fast pace, models are still prone to generating text displaying inconsistencies and factual errors, and NLG outputs should always be submitted to thorough expert review.

## 5. Developing resources and standards for humanitarian NLP

### 5.1. Domain-specific constraints for humanitarian NLP

Creating large-scale resources and data standards that can scaffold the development of domain-specific NLP models is essential to make many of these goals realistic and possible to achieve. In other domains, general-purpose resources such as web archives, patents, and news data, can be used to train and test NLP tools. This, however, does not apply to the humanitarian sector.

Humanitarian technology operates in a context characterized by a set of important domain-specific constraints. NLP technologies should, in fact: (a) satisfy rather strong ethical requirements; (b) support low-resource languages; (c) support deployment by non-experts in real-world contexts, which mandates a fair degree of interpretability. Furthermore, analytical tools and predictive models for humanitarian data require in-depth domain knowledge of the constraints and goals of humanitarian action, as well as of technical aspects of humanitarian assessment and response. The lack of datasets, models, and standards that satisfy these requirements is one of the main obstacles to progress in humanitarian NLP.

The Data Entry and Exploration Platform (DEEP) is an initiative that aims to address these gaps. To date, DEEP is the only large-scale initiative specifically supporting the development of public resources for humanitarian NLP. In the following section, we will therefore introduce the platform, and present one of its most notable derivatives: HumSet, a multilingual dataset annotated using a newly developed unified ontology for humanitarian text.

### 5.2. The DEEP: A platform for collaborative data analysis and resource creation

The Data Entry and Exploration Platform (DEEP^26^) is an initiative that originates from the need to establish a framework for collaborative analysis of humanitarian text data. DEEP provides a collaborative space for humanitarian actors to structure and categorize unstructured text data, and make sense of them through analytical frameworks^27^.

Through DEEP, humanitarians can upload and share their own text data or create collections of existing shared data; annotate them manually or through AI-assisted tools; analyze patterns in the data through customizable analytical workflows; and monitor trends in a dynamic fashion—e.g., to detect changes that may reflect significant increases in humanitarian risk. Through this functionality, DEEP aims to meet the need for common means to compile, store, structure, and share information using technology and implementing sound ethical standards^28^. Importantly, its widespread adoption has made it possible to collect vast amounts of humanitarian text data which can support the development of advanced NLP workflows, as well as expert annotations, which form the backbone of HumSet a standardized ontology that can be used, among other purposes, to train and benchmark domain-specific predictive models for humanitarian text data.

We will present HumSet in the next section, but before describing this as a concrete example of how the platform is contributing to filling important gaps in data and standards for humanitarian NLP, it is worth highlighting that DEEP has already had a profound impact on analytical practices in the humanitarian sector. One of its main sources of value is its broad adoption by an increasing number of humanitarian organizations seeking to achieve a more robust, collaborative, and transparent approach to needs assessments and analysis^29^. DEEP has successfully contributed to strategic planning through the Humanitarian Programme Cycle in many contexts and in a variety of humanitarian projects and initiatives.

For example, DEEP partners have directly supported secondary data analysis and production of Humanitarian Needs Overviews (HNO) in four countries (Afghanistan, Somalia, South Sudan, and Sudan). Furthermore, the DEEP has promoted standardization and the use of the Joint Intersectoral Analysis Framework^30^. The platform has been used to enhance the quality of data analysis in support of strategic and operational planning for different crises, including sudden onset emergencies in Mozambique and Pakistan, complex emergencies (Libya, Nigeria, Yemen, Ukraine), and protracted situations such as civil unrest and population movement from Venezuela. In those countries, DEEP has proven its value by directly informing a diversity of products necessary in the humanitarian response system (Flash Appeals, Emergency Plans for Refugees, Cluster Strategies, and HNOs).

### 5.3. HUMSET: A unified ontology for humanitarian NLP

As anticipated, alongside its primary usage as a collaborative analysis platform, DEEP is being used to develop and release public datasets, resources, and standards that can fill important gaps in the fragmented landscape of humanitarian NLP. The recently released HumSet dataset (Fekih et al., 2022) is a notable example of these contributions. HumSet is an original and comprehensive multilingual collection of humanitarian response documents annotated by humanitarian response professionals through the DEEP platform. The dataset contains approximately 17,000 annotated documents in three languages (English, French, and Spanish) and covers a variety of humanitarian emergencies from 2018 to 2021 related to 46 global humanitarian response operations.

**Figure 3:**
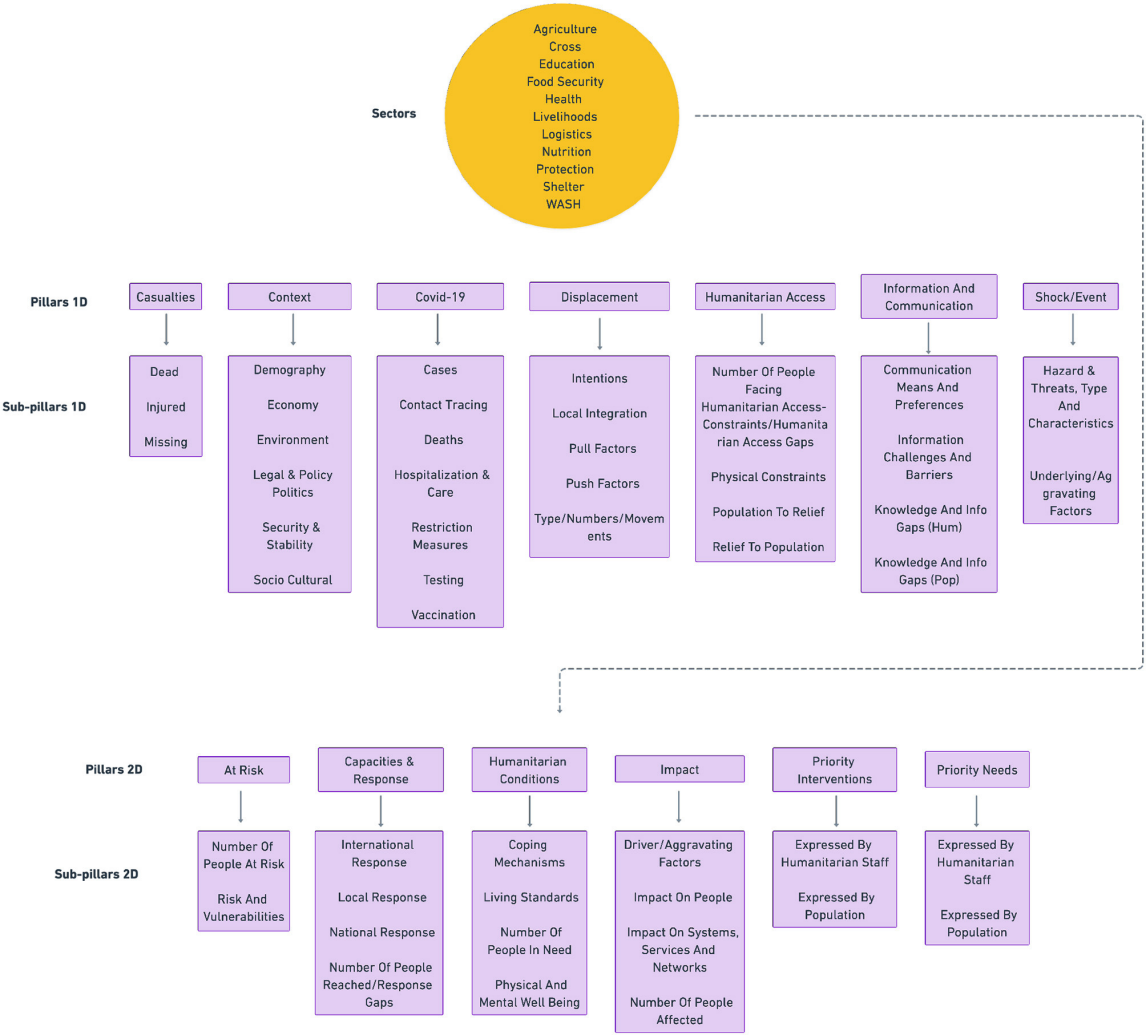
Humanitarian analytical framework developed from DEEP annotations, and used to annotate text in a multidimensional and hierarchical fashion in HumSet (Fekih et al., 2022).

Given that a substantial amount of humanitarian data exists in the form of unstructured text—such as reports, news, and other forms of text data—it is crucial to extract key information and organize it according to sets of pre-defined domain-specific structures and guidelines known as *analytical frameworks*. These analytical frameworks^31^ are the way in which humanitarian organizations and analysts structure their thinking and make the qualitative analysis of data more systematic, and they consist of a hierarchical set of categories or labels with which to annotate and organize data. The HumSet dataset contains the annotations created within 11 different analytical frameworks, which have been merged and mapped into a single framework called *humanitarian analytical framework* (see Figure 3).

**Figure 4:**
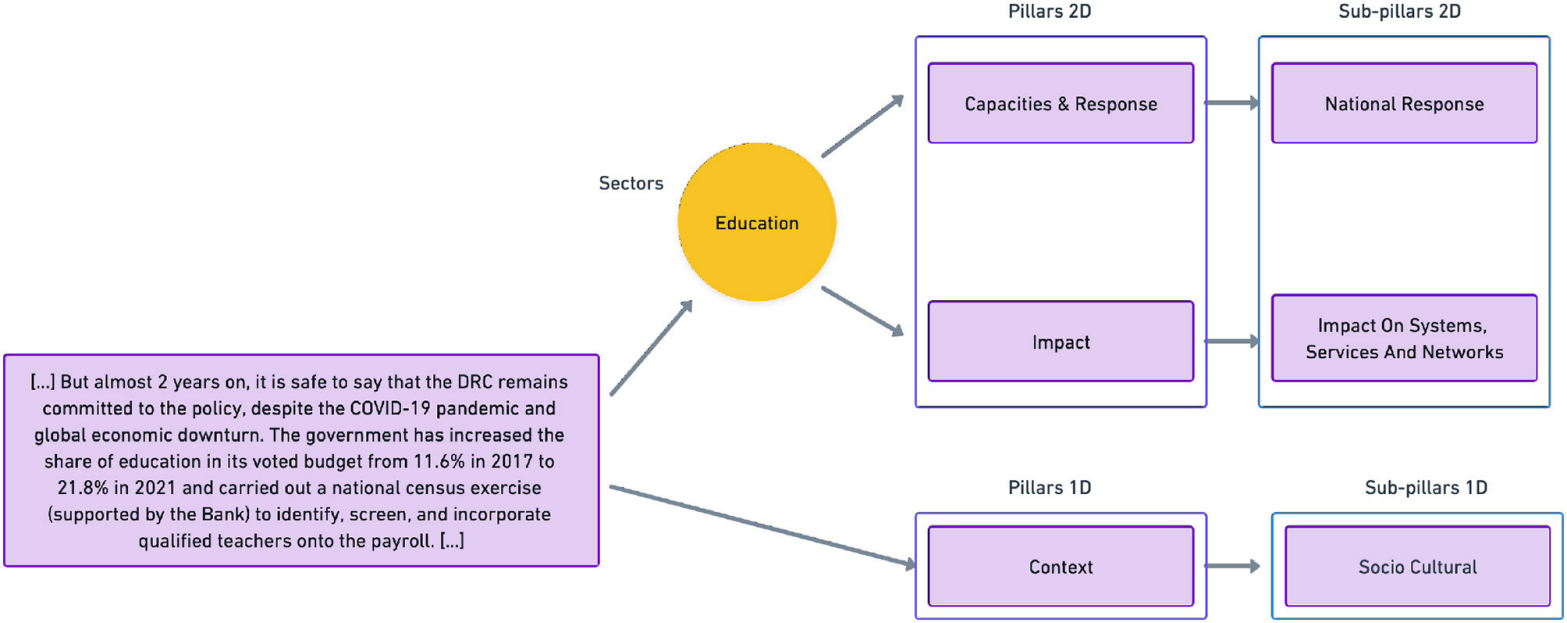
Example of text excerpt from HumSet annotated through its multidimensional and hierarchical ontology (Fekih et al., 2022).

The unified framework has been developed by domain experts and by the creators of the source frameworks through a fully manual process. The process entailed merging and mapping labels from source frameworks to generate a structure that included both overlapping and framework-specific categories. The framework, which can be used to systematically annotate humanitarian text, is composed of three categories of labels (see Figure 4 for a concrete example): Sectors, Pillars/Sub-pillars 1D, and Pillars/Sub-pillars 2D. Pillars and Sub-pillars are hierarchically related (each Pillar is related to a corresponding set of possible Sub-pillars). As the name indicates, 2D Pillars and Subpillars allow for two-dimensional annotations, where, in addition to the Pillars and relative Sub-pillar, text is labeled along one or multiple Sector labels.

HumSet makes it possible to develop automated NLP classification models that support, structure, and facilitate the analysis work of humanitarian organizations, speeding up crisis response, and detection. More generally, the dataset and its ontology provide training data for general purpose humanitarian NLP models. Fekih et al. (2022) presents a set of initial studies analyzing the effect of fine-tuning large pre-trained language models on HumSet text-label pairs, particularly focusing on the task of extracting informative humanitarian text excerpts and classifying them according to the humanitarian analytical framework. The evaluation results show the promising benefits of this approach, and open up future research directions for domain-specific NLP research applied to the area of humanitarian response.

Importantly, HumSet also provides a unique example of how qualitative insights and input from domain experts can be leveraged to collaboratively develop quantitative technical tools that can meet core needs of the humanitarian sector. As we will further stress in Section 7, this cross-functional collaboration model is central to the development of impactful NLP technology and essential to ensure widespread adoption.

## 6. Bottlenecks to adoption and possible solutions

### 6.1. Technical obstacles to large-scale adoption

Despite the value of these initiatives and a growing interest in data-driven humanitarian response, the use of NLP technology in the humanitarian sector is still very sparse. Most projects are never implemented beyond the pilot level, and NLP tools are rarely integrated into decision-making processes in a systematic way. When NLP technology is deployed in the sector, it is often out-of-the-box (commercial) tools and third-party services that are not evaluated on nor tuned to the specific target application domain, and that lack the degree of transparency and control needed for humanitarian applications. The reasons for this arguably include limited awareness of the potential of NLP techniques, an issue which we have touched upon in the previous sections, as well as technical and ethical limitations of current technologies. In the next sections, we will focus on the latter two. What are the main technical bottlenecks to progress, and how can NLP experts, together with humanitarians, leverage ideas, and resources from neighboring fields to overcome them?

Technical challenges can be grouped into three main categories: the need for more domain-specific resources for training and benchmarking, an issue we have partly discussed in previous sections; the need for better multilingual technology; the need for better techniques supporting explainability and interpretation of the behavior of complex models.

### 6.2. Datasets, benchmarks, and multilingual technology

Training state-of-the-art NLP models such as transformers through standard pre-training methods requires large amounts of both unlabeled and labeled training data. Releasing large, open-source collections of reports, news media articles, social media texts, and other secondary sources will be needed to train foundation models for humanitarian NLP, along the lines of existing BERT-like architectures for other domain-specific applications (e.g., SciBERT, see Beltagy et al., 2019).

Sufficiently large datasets, however, are available for a very small subset of the world's languages. This is a general problem in NLP, where the overwhelming majority of the more than 7,000 languages spoken worldwide are under-represented or not represented at all. Languages with small speaker communities, highly analog societies, and purely oral languages are especially penalized, either because very few written resources are produced, or because the language lacks an orthography and no resources are available at all. This can also be the case for societies whose members *do* have access to digital technologies; people may simply resort to a second, more “dominant” language to interact with digital technologies. Developing methods and models for low-resource languages is an important area of research in current NLP and an essential one for humanitarian NLP. Existing work has explored solutions based on massive multilingual model training (e.g., for machine translation, Costa-jussà et al., 2022), on warmstarting and modular retraining of model weights (e.g., Minixhofer et al., 2022), and, for static vector models, on automatic cross-lingual alignment (Thompson et al., 2020). Research on model efficiency is also relevant to solving these challenges, as smaller and more efficient models require fewer training resources, while also being easier to deploy in contexts with limited computational resources.

There have been a number of community-driven efforts to develop datasets and models for low-resource languages which can be used a model for future efforts. The Masakhané initiative (Nekoto et al., 2020) is an excellent example of this. Masakhané aims at promoting resource and model development for African languages by involving a diverse set of contributors (from NLP professionals to speakers of low-resource languages) with an open and participatory philosophy. We have previously mentioned the Gamayun project, animated by similar principles and aimed at crowdsourcing resources for machine translation with humanitarian applications in mind (Öktem et al., 2020).

Developing labeled datasets to train and benchmark models on domain-specific supervised tasks is also an essential next step. Expertise from humanitarian practitioners and awareness of potential high-impact real-world application scenarios will be key to designing tasks with high practical value.

### 6.3. Explainability, bias, and ethics of humanitarian data

Limiting the negative impact of model biases and enhancing explainability is necessary to promote adoption of NLP technologies in the context of humanitarian action. Awareness of these issues is growing at a fast pace in the NLP community, and research in these domains is delivering important progress.

An additional set of concerns arises with respect to ethical aspects of data collection, sharing, and analysis in humanitarian contexts. Text data may contain sensitive information that can be challenging to automatically identify and remove, thus putting potentially vulnerable individuals at risk. One of the consequences of this is that organizations are often hesitant around open sourcing. This is another major obstacle to technical progress in the field, as open sourcing would allow a broader community of humanitarians and NLP experts to work on developing tools for humanitarian NLP. The development of efficient solutions for text anonymization is an active area of research that humanitarian NLP can greatly benefit from, and contribute to.

Collaborations between NLP experts and humanitarian actors may help identify additional challenges that need to be addressed to guarantee safety and ethical soundness in humanitarian NLP. As we have argued repeatedly, real-world impact can only be delivered through long-term synergies between humanitarians and NLP experts, a necessary condition to increase trust and tailor humanitarian NLP solutions to real-world needs.

## 7. Toward a humanitarian NLP community

Both technical progress and the development of an overall vision for humanitarian NLP are challenges that cannot be solved in isolation by either humanitarians or NLP practitioners. Understanding which humanitarian problems could be better addressed with the support of NLP technology requires humanitarians, who are familiar with needs and challenges of humanitarian action, to interact with NLP professionals, who are aware of concrete opportunities and constraints of existing technology. Even for seemingly more “technical” tasks like developing datasets and resources for the field, NLP practitioners and humanitarians need to engage in an open dialogue aimed at maximizing safety and potential for impact.

For these synergies to happen it is necessary to create spaces that allow humanitarians, academics, ethicists, and open-source contributors from diverse backgrounds to interact and experiment. Experiences such as DEEP, Masakhané, and HuggingFace's BigScience project^32^ have provided evidence of the large potential of approaches that emphasize team diversity as a foundational factor, and welcome contributors from diverse cultural and professional backgrounds. As highlighted in Section 5.3, the development of HumSet also provides a concrete example of how qualitative insights contributed by domain experts can be fruitfully combined with quantitative insights to deliver tools that can address the concrete operational needs of humanitarian organizations.

How does one go about creating a cross-functional humanitarian NLP community, which can fruitfully engage in impact-driven collaboration and experimentation? Experiences such as Masakhané have shown that independent, community-driven, open-source projects can go a long way. For long-term sustainability, however, funding mechanisms suitable to supporting these cross-functional efforts will be needed. Seed-funding schemes supporting humanitarian NLP projects could be a starting point to explore the space of possibilities and develop scalable prototypes.

Participatory events such as workshops and hackathons are one practical solution to encourage cross-functional synergies and attract mixed groups of contributors from the humanitarian sector, academia, and beyond. In highly multidisciplinary sectors of science, regular hackathons have been extremely successful in fostering innovation (Craddock et al., 2016). Major NLP conferences also support workshops on emerging areas of basic and applied NLP research.

There are other, smaller-scale initiatives that can contribute to creating and consolidating an active and diverse humanitarian NLP community. Compiling and sharing lists of educational resources that introduce NLP experts to the humanitarian world—and, vice versa, resources that introduce humanitarians to the basics of NLP—would be a highly valuable contribution. Similarly, sharing ideas on concrete projects and applications of NLP technology in the humanitarian space (e.g., in the form of short articles) could also be an effective way to identify concrete opportunities and foster technical progress.

## 8. Conclusions

Current NLP tools make it possible to perform highly complex analytical and predictive tasks using text and speech data. This opens up vast opportunities for the humanitarian sector, where unstructured text data from primary and secondary sources (e.g., interviews, or news and social media text) often encodes information relevant to response planning, decision-making and anticipatory action.

In this paper, we have provided an introduction to the emerging field of humanitarian NLP, identifying ways in which NLP can support humanitarian response, and discussing outstanding challenges and possible solutions. We have also highlighted how long-term synergies between humanitarian actors and NLP experts are core to ensuring impactful and ethically sound applications of NLP technologies in humanitarian contexts. We hope that our work will inspire humanitarians and NLP experts to create long-term synergies, and encourage impact-driven experimentation in this emerging domain.

## Author contributions

RR and NR conceived and designed the structure of this paper. RR wrote the first draft of the manuscript and harmonized individual contributions. NR revised the first draft and coordinated contributions from co-authors. NT, SF, and XC wrote specific sections of the manuscript. All authors contributed to the article and approved the submitted version.

## Footnotes

^1^ https://spacy.io/

^2^ https://thedeep.io

^3^For a deep dive into present-day NLP, including in-depth overviews of the tasks mentioned in the current section, see the 3rd and latest edition of the classic NLP handbook by Jurafsky and Martin (2000). A prepublication draft is currently available at: https://web.stanford.edu/~jurafsky/slp3/.

^4^For a review of neural approaches to NLP preceding the transformer revolution, see Ruder (2018a).

^5^ https://huggingface.co/docs/transformers/model_doc/bloom

^6^see https://www.humanitarianresponse.info/en/programme-cycle/space/page/assessments-overview

^7^ https://centre.humdata.org/

^8^ https://data.humdata.org/

^9^ https://hxlstandard.org/

^10^ https://www.ushahidi.com/

^11^ https://www.openstreetmap.org/

^12^Newer channels like TikTok, and P2P social media like Whatsapp currently do not have public APIs. Furthermore, existing APIs (e.g., Twitter) often impose rate limits or only make available sampled content. Due to these constraints and to technical bottlenecks in interacting with APIs, many users rely on third-party services which provide access to APIs as a service.

^13^ https://lacunafund.org/

^14^ https://lacunafund.org/datasets/language/

^15^ https://www.internal-displacement.org/monitoring-tools/monitoring-platform

^16^ https://www.healthmap.org/en/

^17^ https://www.who.int/initiatives/eios

^18^ https://www.kobotoolbox.org/

^19^ https://www.unicef.org/innovation/U-Report

^20^see e.g., (https://www.digitalhumanitarians.com/chatbots-in-the-humanitarian-field-concepts-uses-and-shortfalls)

^21^ https://commonvoice.mozilla.org/en

^22^ https://tatoeba.org/en/

^23^ https://lanfrica.com/

^24^ https://allenai.github.io/scispacy

^25^ https://www.linkedin.com/posts/data-friendly-space_hno-generated-using-natural-language-processing-activity-6934426752811175936-p3PK?utm_source=li_share&utm_content=feedcontent&utm_medium=g_dt_web&utm_campaign=copy

^26^ https://thedeep.io

^27^A more in-depth description of analytical frameworks is provided in Section 5.3.

^28^DEEP implements ethical standards for humanitarian data science following the DSEG Ethical Framework https://www.hum-dseg.org/sites/g/files/tmzbdl1476/files/2020-10/Framework%20for%20the%20ethical%20use.pdf and the OCHA data responsibility guidelines https://centre.humdata.org/data-responsibility/.

^29^DEEP is governed by a multi-stakeholder consortium that constitutes a cross-section of humanitarian actors, including international NGOs, UN agencies and the RCRC Movement. The current members of the DEEP Governance Board are IDMC, IFRC, iMMAP, OHCHR, Okular-Analytics, UNHCR, UNICEF, UNDP, and UNOCHA. A secretariat is established and hosted by Danish Refugee Council (DRC). The technical platform is developed and maintained by Data Friendly Space INGO (DFS).

^30^ https://www.jiaf.info/

^31^ https://deephelp.zendesk.com/hc/en-us/articles/360006969651-What-is-an-Analytical-Framework-

^32^ https://bigscience.huggingface.co/
